# Microbicides for the Treatment of Sexually Transmitted HIV Infections

**DOI:** 10.1155/2014/352425

**Published:** 2014-02-12

**Authors:** Onkar Singh, Tarun Garg, Goutam Rath, Amit K. Goyal

**Affiliations:** Department of Pharmaceutics, ISF College of Pharmacy, Moga, Punjab 142001, India

## Abstract

Approximately 34 million people were living with human immunodeficiency virus (HIV-1) at the end of 2011. From the last two decades, researchers are actively involved in the development of an effective HIV-1 treatment, but the results intended are still doubtful about the eradication of HIV. The HIV-1 virus has gone from being an “inherently untreatable” infectious agent to the one liable to be affected by a range of approved therapies. Candidate microbicides have been developed to target specific steps in the process of viral transmission. Microbicides are self-administered agents that can be applied to vaginal or rectal mucosal surfaces with the aim of preventing, or reducing, the transmission of sexually transmitted infections (STIs) including HIV-1. The development of efficient, widely available, and low-cost microbicides to prevent sexually transmitted HIV infections should be given high priority. In this review, we studied the various forms of microbicides, their mechanism of action, and their abundant approaches to control the transmission of sexually transmitted infections (STIs).

## 1. Introduction

**Figure 1:**
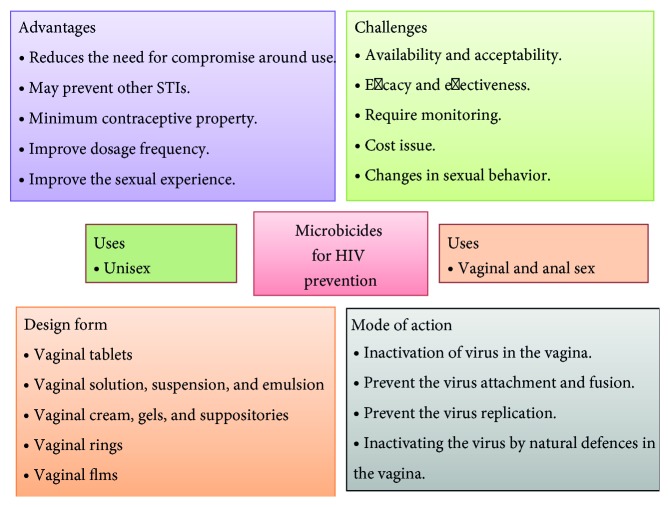
An overview of microbicides for HIV prevention.

According to estimates by World Health Organization (WHO) and United Nations Acquired Immunodeficiency Syndrome (UNAIDS), 34 million people were living with HIV at the end of 2011. In that same year, some 2.5 million people became newly infected, and 1.7 million died of AIDS-related causes, including 2, 30,000 children [1]. More than 95% of all new HIV infections are found in developing countries, out of which 87% were acquired through heterosexual transmission. Heterosexual transmission of HIV is an important factor in driving the current major epidemic in Africa, India, and China [2]. Heterosexual transmission is influenced by various epidemiological factors such as age, gender, mobility, and the presence of other sexually transmitted infections. A significant characteristic of heterosexual transmission is the inconsistent burden of HIV infection in women compared to men. Women are two to four times more susceptible than men to acquire sexually transmitted HIV infection [3]. Current options to reduce transmission and acquirement of HIV infection remain limited for women. There is a clear need for new technologies to prevent the sexual transmission for the same. Making accurate and steady use of male condoms has been shown to prevent HIV transmission, but women often are unable to negotiate the use of condoms by their male partners. The female condom, alternative barrier method which is not preferable due to high cost, requires a certain level of skill and requires acceptance by the male partner [4]. So, need of new technologies to control the risk of sexual HIV acquisition. Microbicides are self-administered prophylactic agents that could be applied to the vagina or rectum to protect against sexually transmitted infections (STIs) including HIV. They can be formulated as gels, creams, films, or suppositories to reduce their risk of sexual acquisition of HIV. Microbicides may or may not have spermicidal activity (contraceptive effect) [5]. In this review, we studied the various forms of microbicides, their mechanism of action, and their abundant approaches to control the transmission of STIs (Figure 1).

## 2. History of Microbicides Development

Microbicides were originally conceived as products by using simple nondrug-based compounds. It was provided over-the-counter (OTC) without a prescription along with broad protection against all or most STIs [6]. The condom is known to offer good protection against HIV and other STIs. Now one question arises in the mind that why one requires microbicides to produce anti-HIV effects? Condoms are successful against these infections when they are used every time and correctly. Intensive campaigns to promote condoms remain as important as ever and there have been some successes. But they are not liked in many of the societies in which HIV is prevalent, because women lack the power to negotiate their use. By contrast, microbicides will provide an accessible technology that will broaden the range of protective options and, more importantly, will be under the control of women [7]. There is a prediction that they will be widely acceptable to both men and women. Unlike condoms, microbicides will not create a physical barrier to intimate contact, and women will be able to apply them for a considerable time before intercourse. Ideally, microbicides will be colorless, odorless and tasteless, and nonmessy to use. Moreover, the use of a microbicide need not necessarily prevent conception, as the intention is to develop both contraceptive and noncontraceptive products. To be a microbicide, these agents must have the safety and effectiveness following vaginal or rectal administration and should cause minimal or no genital symptoms after repeated usage. A safe and efficacious anti-HIV microbicide is not yet available, despite the fact that more than 60 candidate agents have been identified which have *in vitro* activity against HIV and five products have undergone large scale phase III trials such as nonoxynol-9, savvy, cellulose sulphate, carraguard, and PRO 2000. Clinical development of nonoxynol-9 was terminated because there was evidence that it increased susceptibility to HIV infection [8]. Various important key events and predictions in the development of microbicides are shown in Table 1.

## 3. Mechanism of Microbicides Actions

**Figure 2:**
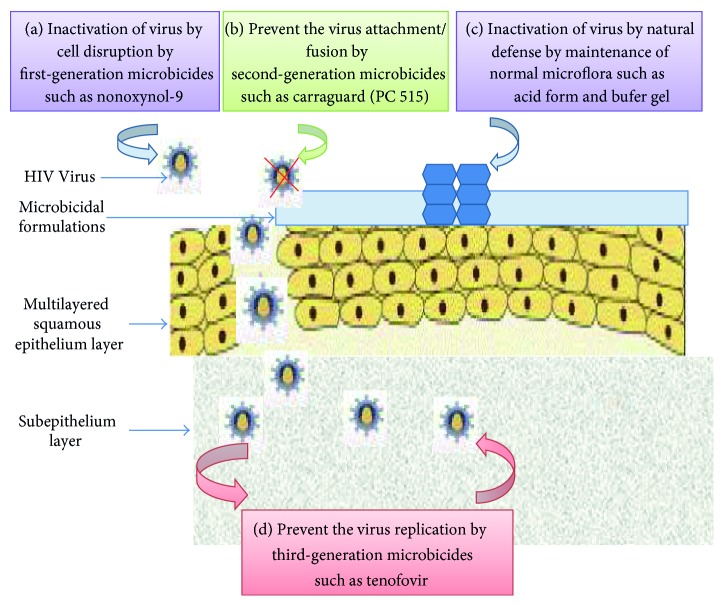
Mechanism of action of microbicidal formulations.

The principal target of microbicides is to reduce or prevent both male-to-female and female-to-male HIV transmission. The only microbicide with proven efficacy is tenofovir gel which has been shown to protect against HIV. Subpopulation analyses from trials of some products have shown trends towards effectiveness against other STIs, but none have been proven in full phase III efficacy trials. Now, it becomes the major challenge for our researchers to know the causes for failure and improve its efficacy against STIs as well as other infections.  Table 2 represents the various generations of microbicide candidates along with their mechanism of action. To prevent HIV infection, microbicides must cover one or more of the following mechanisms which are shown in Figure 2.

### 3.1. Inactivation of Virus in the Vagina

First-generation microbicide candidates such as nonoxynol-9, savvy, and sodium lauryl sulphate inactivate the vaginal virus by disrupting cell membranes or changing the cell membrane structure, which make it more porous and more liable to disruption [40].

#### 3.1.1. Nonoxynol-9

Nonoxynol-9 (nonylphenoxypolyethoxyethanol or N-9) was the first spermicidal agent, to be evaluated for effectiveness against HIV transmission. It is available over-the-counter in different formulations such as gels, suppositories, and film. According to the Centers for Disease Control and Prevention (CDC) and the World Health Organization (WHO), it is a safe and effective contraceptive option for women at low risk for HIV/STIs, but the FDA recently proposed a change in labeling for nonoxynol-9 products to inform consumers that nonoxynol-9 does not offer protection against HIV/STIs12 [41].

#### 3.1.2. Savvy

C31G (savvy) (contains cetyl betaine and myristamine oxide) is an antimicrobial and spermicidal agent. Its primary mechanism of action acts as a surfactant, which diffuses through cervical mucus more rapidly than nonoxynol-9. At low concentrations, it is not toxic to vaginal cells. C31G kills sperm cells and a variety of STI pathogens, including HIV [42]. But after various studies, savvy was shown to offer no protection against HIV infection in phase III trials, and in one trial showed a trend towards risk of infection. Consequently, development of savvy was terminated 5 or 6 years ago.

#### 3.1.3. Sodium Lauryl Sulphate

A surfactant compound that has been shown to disrupt both enveloped and nonenveloped viruses. This agent has been formulated to act as an invisible condom. It can cover the vaginal wall as a liquid at room temperature, then transform into a gel at body temperature, and block HIV-1 and STI transmission. Safety of sodium lauryl sulphate has been shown in at least two phases clinical trials [43].

### 3.2. Prevent the Virus Attachment and Fusion in the Genital Tract

Second-generation microbicide candidate prevents the virus attachment and fusion in the genital tract by blocking viral entry into susceptible cells via blocking CD4 attachment or interfering with attachment to host cells. Many of these products are nonspecific blockers. They act against multiple organisms, including microbes that cause HIV and other STIs [44].

#### 3.2.1. Polynaphthalene Sulphonate (PRO 2000)

Polynaphthalene sulphonate binds to HIV and other STI pathogens which provide protection from infection to human cells. Phase I clinical trials have expressed the safety and acceptability of PRO 2000 in low doses, although one-third to two-third of women experienced mild vulval irritation or leakage of the microbicide from the vagina [45]. Similar to savvy, it also failed to demonstrate efficacy in phase III trials and is no long being developed.

#### 3.2.2. Carraguard (PC-515)

Carraguard (containing carrageenan) microbicidal gel successfully binds to viruses (HIV, HPV, and herpes simplex virus) and blocks the pathway from sticking to healthy cell. Carrageenan is commonly used in cosmetics, toothpastes, and food due to safe and nontoxic nature. In clinical trials, carraguard is distributed in prefilled, single-dose, disposable plastic applicators [46]. Carraguard also fails to demonstrate efficacy in phase III trials and is no long being developed.

#### 3.2.3. Cellulose Sulfate

Polyanions cellulose sulfate is a long chain sulfated polysaccharide that is being developed as a contraceptive and microbicidal agent. It has an *in vitro* activity against a broad spectrum of STI pathogens and shown inhibitory effect up to 8 h after initiation of infection. Phase 1 safety studies have been conducted in HIV-1 infected seronegative and seropositive women, and data suggested that it is safe and well tolerated [47]. But it showed no protection against HIV infection in phase III trials and showed a trend towards increased risk of infection.

### 3.3. Inactivating the Virus by Natural Defences in the Vagina

Microbicide candidates could be used to supplement or enhance the natural immune defences of the vagina. The vagina is normally too acidic for sperm to survive, evolving to provide a hostile environment for pathogens including HIV. A crucial feature of this environment is the ability of resident lactobacilli (naturally in the vagina) to maintain an acidic pH. Lactobacilli release a variety of antimicrobial compounds such as lactic acid, hydrogen peroxide, bacteriocins, and biosurfactants. Semen, having an alkaline pH, neutralizes the acidity of the vagina, making the easily survival of sperm, HIV, and other pathogens [48]. Bacterial vaginosis is a condition that increases the risk of HIV infection by diminishing lactobacilli, so vaginal pH rises, and these changes appear to result in increased susceptibility to HIV transmission. Semen, having alkaline pH, is made acidic by acid-buffering microbicides, which keeps the vagina acidic, thus inactivating sperm and several sexually transmitted organisms, including HIV. Microbicides are being developed that maintain an acidic pH (buffer gel and acid form) or that replace absent lactobacilli (*Lactobacillus crispatus*) [49].

#### 3.3.1. Acid Form

This is an acidic buffering gel that is approved as a sexual lubricant or as a spermicidal microbicide. This new delivery system was designed to maintain the acidic vaginal milieu (the low pH inactivates many pathogens and spermatozoa), form a protective layer over the vaginal/cervical epithelium (minimizing contact with pathogenic organisms), and provide long-term vaginal retention. The acid buffering gel has been reported to be effective against herpes simplex virus, chlamydia, gonorrhoea, papillomavirus, and HIV-infected leukocytes and provide dual protection from pregnancy and sexually transmitted infection [50].

#### 3.3.2. Buffer Gel

Buffer gel is a buffering carbopol polymer that has spermicidal activity, keeps the vagina acidic even in the presence of semen, and creates a physical barrier that stops or slows down the passage of pathogens into the vaginal and cervical walls. Preclinical testing has shown that buffer gel can inhibit pregnancy, HIV transmission, human papilloma virus (HPV), herpes simplex virus (HSV), and chlamydia infections without damaging the reproductive epithelium or microflora [51]. But development of buffer gel was terminated because it failed to show efficacy in phase III trials.

### 3.4. Prevent the Virus Replication

Third-generation microbicides candidate acts locally in the reproductive tract mucosa at specific steps in the HIV replication cycle and inhibits the replication process of virus. It has a narrow spectrum of activity against HIV. Reverse transcriptase inhibitors act by inhibiting HIV-1 viral reverse transcriptase, a critical enzyme required for the conversion of viral RNA into DNA before its integration into the host genome. In the presence of reverse transcriptase inhibitors, this process is suppressed thus resulting in quantitative reductions in viral replication. Various advantages of the reverse transcriptase inhibitor class of microbicides include available various numbers of marketed products, and cost effective production without emergence of antiretroviral resistance is associated with use of reverse transcriptase inhibitor [52].

#### 3.4.1. Tenofovir

Activation of tenofovir (9-[(R)-2-(phosphonomethoxy) propyl] adenine monohydrate, or PMPA) is reliant upon anabolic phosphorylation by intracellular nucleoside kinases, whose activity and availability are dependent upon the activation state of the cell. Tenofovir disoproxil fumarate (oral prodrug of tenofovir) is currently under appraisal as an oral agent for the prevention of HIV infection in high-risk populations [19]. Tenofovir disoproxil fumarate in combination with emtricitabine (truvada) has now been approved by the FDA for prevention.

#### 3.4.2. Zidovudine

The first drug to be approved for treatment of HIV infection was zidovudine. It is available in different dosage forms such as capsule, tablet, and syrup and in intravenous form. The main mechanism is suppressing HIV viral load and increasing CD4 cell counts as well as acting as protease inhibitor or nonnucleoside reverse transcriptase inhibitor. It also shows dramatic reduction in mother-to-child transmission of HIV in previously untreated mothers [53].

#### 3.4.3. Stavudine

Stavudine was approved to use in combination therapy for HIV infection due to evidence of efficacy such as sustained increases in CD4 cell counts and reductions in HIV viral load. Stavudine is available in capsule or solution formulations; these are approved for twice-daily dosing. Stavudine and zidovudine should not be used in grouping because they strive with each other for stimulation by intracellular phosphorylation, resulting in diminished antiviral activity [54].

## 4. Formulation Approach for Microbicide Development

Vaginal drug delivery systems include a large variety of pharmaceutical forms such as semisolids, tablets, vaginal films, and vaginal rings. Vaginal microbicides are self-administered products that can be applied to vaginal mucosal surfaces with the goal of preventing or significantly reducing the transmission of STIs including HIV-1 [55]. The vaginal dome is subject to conditions that make it a target for disease and infection during sexual intercourse. Treatment for the prevention of conception and treatment of STDs usually involves the systemically or topically delivery of active agents to the vaginal dome [56]. An ideal microbicide should have an established safety record and lack genital epithelial toxicity. Microbicides would provide protection from inactivating microbes or by preventing microbes from replicating either in semen or in the infected host cells lining the vaginal wall. The emergence of HIV/AIDS as a disease spread through sexual intercourse and the problems associated with other viral STDs has asked the searchers for new, effective, acceptable, and safe vaginal microbicides for curbing mucosal and viral transmission [6]. Vaginal mucoadhesive preparations have been developed as a new type of controlled release form for the treatment of both topical and systemic diseases. For drugs that are susceptible to gut or hepatic metabolism or which cause GI side effects, vaginal delivery may offer a number of advantages over the other routes of administration. The greatest advantage of such dosage forms is the possibility of maintaining them in the vagina for extended periods of time including daytime and nighttime, thereby enabling lower dosing frequencies. The vagina is a fibromuscular tube connecting the uterus to the exterior of the body [57]. The surface area of the vagina is increased by numerous folds in the epithelium and by microridges covering the epithelial cell surface. Among the polymers, polyacrylic acid and hydroxypropyl methyl cellulose are the ideal excipients in mucoadhesive strength. In general, traditional vaginal dosage forms include solutions, suspensions, gels, microparticles, suppositories, creams, foams, and tablets and all have a relatively short contact time [58]. Various important characteristics such as safety, efficacy, cost, acceptability, efficiency, drug delivery pattern, and resistance mainly affect the development of microbicide. Ideal characteristics for microbicide development [59] are shown in Table 3.

## 5. Mucoadhesive Polymers Used for Microbicide Fabrication

Mucoadhesive polymers have gained interest among pharmaceutical scientists as a means of improving drug delivery by promoting residence time with the mucous membranes. Mucoadhesion is defined as a process in which two materials (biological in nature) are held together for an extended period of time by interfacial forces. The first step in the process of mucoadhesion involves an intimate contact between a mucoadhesive polymer and a membrane [60]. In the second step, after contact is established, penetration of the mucoadhesive into the crevice of the tissue surface or interpenetration of the chains of the mucoadhesive with those of the mucus takes place. The three important mechanisms behind the mucoadhesion are (1) polymers becoming sticky and mucoadhesive after the contact with water, (2) polymers adhering through nonspecific noncovalent interactions, and (3) polymers binding to specific receptor site [61].

### 5.1. Ideal Characteristics of Mucoadhesive Polymer [26, 57, 62]

- It must be loaded substantially by the active compound.
- The polymer as well as its degradation products should not cause toxicity.
- It should be nonirritant to the mucous membrane.
- It should preferably form a strong noncovalent bond with the mucin-epithelial cell surfaces.
- It should adhere to most tissue and should possess some site-specificity.
- The polymer must not decompose on storage or during the shelf life.
- It should have high molecular weight.
- It should have strong anionic charges.
- It should have strong hydrogen bonding groups.
- It should have surface tension characteristics suitable for wetting mucosal surface.

Numbers of bioadhesive polymers have been described for different mucosal sites and the polymers which were already in use as pharmaceutical excipients were tested for their mucoadhesive properties. Several groups of polymers have been tested as vaginal delivery systems.

#### 5.1.1. Cellulose Derivatives

The number of cellulose derivatives like hydroxy ethyl cellulose (HEC), hydroxy propyl cellulose (HPC), hydroxy propyl methyl cellulose (HPMC), methyl cellulose (MC), sodium carboxymethylcellulose (Sod CMC), and so forth has been used for intravaginal drug delivery systems. Among all the cellulose derivatives, Sod CMC had excellent mucoadhesive properties. Sodium carboxymethylcellulose is used as mucoadhesive polymer in Gynol II, a contraceptive jelly, having nonoxynol-9 as active substance and is used as a spermicidal contraceptive in conjunction with barrier methods of contraception. HPMC and HEC were also assessed as mucoadhesive polymer in the formation of the bioadhesive vaginal films of sodium polystyrene sulphonate (PSS), a novel contraceptive antimicrobial agent [63].

#### 5.1.2. Carbopol

A high molecular weight polymers of acrylic acid have been used mainly for their mucoadhesive properties in vaginal drug delivery systems. Carbopol polymers are manufactured by cross-linking process. Different grades of carbopol are available depending upon the degree of cross-linking and manufacturing conditions. Each grade is having its significance for its use in pharmaceutical dosage forms. Carbopol 934 P is cross-linked with allyl sucrose and is polymerized in solvent benzene [64]. Carbopol 71G, 971 P, and 974 P are cross-linked with allyl penta erythritol and polymerized in ethyl acetate. Carbopol is used in formulation of the mucoadhesive polymeric films developed as female-controlled drug delivery system (FcDDS). It does not only increase the degree of hydration and mucoadhesiveness of carbopol but also maintain the morphology of the films in the vaginal cavity. The capability of carbopol gel to attach to lymphocytes makes them a site-specific drug delivery system for AIDS prophylaxis. FcDDS in a form of carbopol gel has been developed as an intravaginal delivery system to prevent the onset of STDs. Higher viscous bioadhesive polymer gel enhanced the duration of mucosa contact, while the release rate of a drug molecule from polymer-based gel remained as fast as it remained in water [65].

#### 5.1.3. Polycarbophil

It is a cross-linked polymer in divinyl glycol and polymerized in solvent benzene. Polycarbophil gel is capable of altering vaginal and suburethral blood flow favourably from the surface of the vaginal tissue. It leads to significant improvement in dry vagina and menopause related stress incontinence [12].

#### 5.1.4. Polyacryaltes and Poly (Acrylic Acid)

Acrylate is a family of polymers, which is a type of vinyl polymers. In most of the vaginal preparations, poly (acrylic acid) (PAA) derivatives have been used as mucoadhesive polymer. Polyacryaltes and poly (acrylic acid) have good mucoadhesive strength. Among all PAA derivatives in most of the vaginal preparations, either carbopol or polycarbophil has been used as mucoadhesive polymer [66].

#### 5.1.5. Sodium Alginate

Sodium Alginate is a natural amylose carbohydrate distilled from alga. Sodium alginate consists of sodium salt of alginic acid, which is a mixture of polyuronic acid composed of residue of D-mannuronic and L-guluronic acids. The adhesiveness of the hydrogel of sodium alginate has been explored and it is found that increasing concentration increases the adhesiveness of the polymer [67].

#### 5.1.6. Gelatin

Gelatin is a mixture of purified protein fraction obtained either by partial acid hydrolysis (type A) or by partial alkaline hydrolysis (type B) of animal collagen. Gelatin contains 84–90% protein, 1-2% mineral salts, and 8–15% water and is free from additives and preservatives. The old vaginal dosage forms which were based on gelatine can be seen as the first mucoadhesive systems [68].

#### 5.1.7. Hyaluronic Acid

Sodium hyaluronate is the principal form of hyaluronic acid at physiological pH. It is most common negatively charged glycosaminoglycan. In its natural form, hyaluronic acid exists as a high molecular weight polymer of 106-107 Da. Polymeric systems (microspheres) prepared from hyaluronic esters have been assessed for the vaginal administration of calcitonin for postmenopausal osteoporosis [69].

#### 5.1.8. Carrageenan

Carrageenan is usually derived from red alga, sometimes called Irish moss. It consists of potassium, sodium, calcium, magnesium, and ammonium sulphate esters of galactose and 3, 6-anhydrogalactose copolymer. The carrageenan-based nonoxynol-9 formulation may provide a greater degree of protection from vaginal transmission of HSV-2, HPV, and HIV infections. The trail ran from 2004 to 2007 on more than 4,000 South African women and the result showed that the gel is safe at least, not increasing infection any more than the baseline or causing significant side effects. As such, they believe to use it as an established delivery vehicle for experimental antiretroviral in future studies. The main advantages of carrageenan are safe, cheap, widely available, and effective over a wide pH range. Carrageenan has the ability to retain its properties at high temperatures and remains effective for hours within the vagina [70].

#### 5.1.9. Poly Vinyl Alcohol (PVA)

PVA is water soluble synthetic polymer which is an odorless, tasteless, translucent, white, or cream-colored granular powder. The value of “*n*” for commercially available materials lies between 500 and 5,000, equivalent to a molecular weight (MW) range of approximately 20,000–200,000. In the development of the bioadhesive vaginal films of PSS, they possessed good peel ability, flexibility, and physical as well as mechanical properties [71].

#### 5.1.10. Chitosan

It is a linear polysaccharide which is composed of *β*-(1-4)-linked D-glucosamine (deacetylated unit) and N-acetyl-D-glucosamine (acetylated unit). Chitosan is cationic polyamine with a high charge density at pH < 6.5 so adhere to negatively charged surfaces. Partial deacetylation of chitin results in the production of chitosan, which is a polysaccharide comprising copolymers of glucosamine and N-acetyl glucosamine. Chitosan is commonly used to obtain pharmaceutical systems for mucosal delivery. Chitosan is currently receiving a great deal of attention for medical and pharma applications, particularly because of its penetration enhancement capability, alongside with other intrinsic properties such as biocompatibility, biodegradability, bioadhesivity, and bacteriostatic effects. Chitosan citrate has been used as multifunctional polymer for vaginal delivery. Chitosan citrates possess potential properties of penetration enhancement [72]. By introducing the thiol group on the chitosan, bioadhesiveness of the chitosan could be significantly increased.  Table 4 shows list mucoadhesive polymers along with their bioadhesiveness. The bioadhesiveness of a polymer is affected by the nature of the polymer and by the surrounding media.  Table 5 shows the impact of various factors such as concentrations, molecular weight, pH, and disease state on the bioadhesiveness of a polymer.

## 6. Different Microbicidal Products for Drug Delivery

Vaginal drug delivery systems include a large variety of pharmaceutical forms such as tablets, solutions, suspensions, gels, microparticles, suppositories, creams, foams, vaginal films, and vaginal rings that are successfully use as microbicidal products to prevent or control HIV transmission, human papilloma virus (HPV), and various other types of sexually transmitted infections.

### 6.1. Vaginal Tablets

Vaginal formulations available in Indian market are vaginal tablets. Conventional vaginal tablets accessible around the world consist of anti-infective agents, hormones, plant extracts, and *Lactobacillus* spores. Microbicide candidates formulated as tablets include cellulose sulfate, acid form, polystyrene sulfonate, dapivirine, DS003, tenofovir, and UC781 [73]. Among all of these, cellulose sulfate, polystyrene sulfonate, and UC781 are no longer in development as microbicides. A vaginal microbicide formulation should be easy to apply, available at a reasonable cost, and in a user acceptable form. The main purposes of using vaginal tablet are local delivery at the site of administration (e.g., vaginal, rectal), for systemic absorption, avoidance of first-pass metabolism (oral), patient or care provider convenience, and improved targeting to the site of action (e.g., uterus). A fast dissolve tablet should release the API in such a way that complete dissolution is attained at the site of administration [74]. Other advantages are portability, precise dosing, ease of storage, handling and administration, feasibility of large scale production, and low cost. Tablets also offer the potential for improved stability of drugs at extremes of temperature and humidity. Tablets can be designed with additional characteristics such as bioadhesion, sustained release, and rapid dispersion with the help of specific excipients [75]. Tablet dosage forms can be used to address the issues of leakage and messiness that can be associated with conventional vaginal gel formulations. Praneem polyherbal tablets are an example of a vaginal microbicide tablet which is in phase II clinical trials in India. However, various difficulties face in the development of microbicides tablets as the following.

#### 6.1.1. Lack of a Reference

Microbicides, being one of the new formulations, are not currently official in any pharmacopoeia; thus regulatory standards for evaluation of the formulation are a challenging exercise.

#### 6.1.2. Complexity to Ensure Content Uniformity, Especially with Low Dose Actives

As these tablets are high in weight, ensuring content uniformity of low dose actives can be tough [76].

#### 6.1.3. Complexity in Optimization of the Composition

Disintegration of vaginal tablets should be rapid and form smooth, homogenous, viscous, and bioadhesive dispersions. Achieving these goals may be difficult with tablet compositions containing relatively large amounts of excipients in the vaginal fluid.

#### 6.1.4. Combination Products and Its Compatibility

If formulation contains more than one active drug, establishing the compatibility of the actives of drugs with excipients and establishing a combined method of analysis of the APIs will present more challenges compared with tablets prepared with single API [77].

### 6.2. Vaginal Solutions, Suspensions, and Emulsions

Vaginal solutions, suspensions, and emulsions are liquid preparations intended for a local effect, for irrigation or for diagnostic purposes and prevent the HIV infections. They may contain excipients to adjust the viscosity of the preparation, to adjust or stabilize the pH, to increase the solubility of the active substance(s), or to stabilize the preparation. The weak acid hypochlorous solution (pH 5.0-6.0) had excellent microbicidal effect against various bacteria, fungi, and virus *in vitro*. The weak acid hypochlorous solution had excellent microbicidal effect against a broad microbicidal spectrum of standard strains and clinical isolates in a short time [78, 79]. Vaginal suspensions may show sediment that is readily dispersed on shaking to give a suspension which remains sufficiently stable to enable a homogeneous preparation to be delivered [80]. Vaginal emulsions may show evidence of phase separation but are readily redispersed on shaking. The main advantages of these dosage forms are easiness of administration, possibility of autoadministration, hepatic first-pass effect bypass, low systemic drug exposure (namely, in the case of products used for local conditions), and increased permeability for some drugs when comparing to the oral or other routes. However, it suffers from major limitations including menstrual cycle-associated vaginal changes, genital hygiene issues, local side effects, and variable drug permeability [55].

### 6.3. Vaginal Gels, Cream, Suppositories, and Foam

Vaginal gels, cream, suppository, and foam are a drug delivery system that is inserted into the vagina, where it dissolves or melts [81]. They are used to deliver both systemically-acting and locally-acting medications [28]. The general principle is that the suppository is inserted as a solid and will dissolve or melt inside the body to deliver the medicine which received by many blood vessels that follow the larger intestine. These are generally conical, rod shaped, or wedges shaped and are larger than rectal suppositories (4–8 g). All the above formulations act as barriers to prevent entry of sperm into the female's upper vagina. Spermicides also act like chemicals that kill the sperm [82]. They are commonly used for local actions in the treatment of vaginal infections such as HIV transmission, human papilloma virus (HPV), herpes simplex virus (HSV), and chlamydia infections [83]. Various drug candidates such as miconazole nitrate are successfully delivered in the form of miconazole nitrate vaginal suppositories USP, 200 mg, to control vaginal yeast infections as well as relieving external itching and irritation due to a vaginal yeast infection. The main advantages of these dosage forms are self-administration which is possible avoiding first-pass metabolism, localizing action reduced systemic distribution, and reducing systemic toxicity. However, it suffers from some limitations such as mucosal irritation; installation may trigger defecation reaction, high cost of manufacture, and patient incompliance [84].

### 6.4. Vaginal Rings

Intravaginal rings (IVRs) offer a unique delivery method over other solid and semisolid formations. IVRs are flexible, torus-shaped polymeric devices either loaded with active pharmaceutical ingredients (APIs) within the polymer matrix of the ring or within a reservoir core at the center of the ring. The largest controlling factor of the IVRs is the pliability of the polymer ring backbone. The rings need to allow for compression as they are inserted into the vagina and placed in the upper third of the vagina to prevent involuntary expulsion [85]. The vaginal ring contains drug substances, which were homogenously dispersed throughout the matrix. The drug release from these matrixes is mainly depending upon drug loading as well as surface area of the devices. Three important mechanisms are involved for drug release from above system such as dissolution, diffusion, and partition. Firstly, drug was released at the surface of a matrix ring and produced drug depletion zone. Mostly solubilized drug molecules and other substances must diffuse from the created zone. Based on the nature of polymer or its applications, they are classified into various categories [86].

#### 6.4.1. Silicone Elastomer Vaginal Rings

Silicone elastomer is widely used in medical and pharmaceutical devices due to its biocompatible nature. Numerous forms of silicone elastomer commercially exist with different chemical cross-linking mechanisms. For example, Estring and Femring were manufactured from a two- and three-part addition cure and condensation cure silicone elastomer respectively [87]. This cross-linking prevents initial burst effects, variable release during storage time and do not produce an alcohol as byproduct. The prospective for controlled release of HIV microbicides was first recognized for nonoxynol-9. The dapivirine (nonnucleoside reverse transcriptase inhibitor) has been broadly tested in silicone elastomer vaginal rings [32].

#### 6.4.2. Thermoplastic Vaginal Rings

Thermoplastic elastomers such as poly (ethylene vinyl acetate) and segmented polyurethane (PU) are used for the construction of thermoplastic vaginal rings. Injection molding or continuous hot-melt extrusion technique is mainly used for the fabrication of the same systems. In this technique, the drug is compounded into molten polymer (using melt mixing) and then forced into a mold (by melt extrusion screws). The polymer rod is cut to length and end-joined together (using butt welding) to form the final ring. The contraceptive NuvaRing (10 to 40% vinyl acetate content) is only intravaginal thermoplastic ring, which is currently available in the market [88].

#### 6.4.3. Novel Vaginal Ring Designs

*Multisegment Vaginal Rings.* Multisegment vaginal ring contains attached two or more sections, which was loaded with one or more drug in each separate section. An advantage of multisegment nonisotropic drug delivery device includes controlled release rate, higher entrapment efficiency, prevent drug-drug interactions, and ability to separate the incompatible drug molecules. Multisegment drug delivery devices have the limitation of requirement for expressively additional refined manufacturing system than what is mandatory for distinct segment vaginal rings [89].

*Biosoluble and Hydrogel Vaginal Rings.* Polymer materials such as styrene-butadiene block copolymers, acacia gum, and sodium methacrylate have been considered for fabrication of vaginal rings. Styrene-butadiene block copolymers constructed matrix type rings are successfully used for controlled release of 17-estradiol for treatment of postmenopausal symptoms. These systems are very useful for the sustained as well as controlled delivery of hydrophilic and/or large molecular weight actives.

Acacia gum, 2-hydroxyethyl methacrylate, and sodium methacrylate constructed vaginal ring provide sustained release of combination antiretroviral HIV microbicides for up to 28 days [90].

*Coated Pod-Insert Vaginal Rings.* Drug cores are coated with layers of semipermeable polylactic acid polymer into a silicone elastomer ring. They show pseudozero order release kinetics behavior and release rate determined by the window diameter. The amount of drug released from ring is mainly depending upon amount of polymer coating, composition of polymer coating, drug delivery window size, and nature of the rings [85]. The main advantages of these systems act as a solid dosage inserts, follow controlled release mechanism, stabilize the active molecule, and can deliver peptide as well as protein-based microbicide molecules [91].

### 6.5. Vaginal Films

Film dosage forms are thin strips of polymeric water-soluble substances that get dissolved when placed on the vaginal mucosal surface to release the active ingredient. Thin films are suitable dosage forms which can be administered without an applicator. Other advantages include portability, easy storage, discreet use, no product leakage, and low unit dose cost. Thin films can be used to stabilize drugs vulnerable to degradation in aqueous environments. Film formulations are comprised of the active pharmaceutical ingredient (API), water soluble polymers, plasticizers, fillers, color, and flavor [92]. Polymers used should be nontoxic; nonirritant; devoid of leachable impurities; possess good wetting and spread ability; exhibit sufficient peel, shear, and tensile strength; and inexpensive to manufacture and package. Type of polymer and molecular weight of polymer can appreciably impact properties of the film such as mechanical strength and disintegration time. Thin film formulations include plasticizers to provide flexibility. Film dosage forms are being used as vaginal formulations. Vaginal film product marketed is the vaginal contraceptive Film (VCF), which is having spermicidal agent Nonoxynol-9 (N-9) [93]. Vaginal films have been used as delivery systems for drugs such as antifungal and antibacterial. Vaginal bioadhesive films contained clindamycin phosphate for treatment of bacterial vaginosis (BV). Film dissolution profile and drug release are crucial factors to be considered when applying thin film technology to vaginal microbicide delivery. Additionally vaginal fluids and microflora may have an impact on drug delivery from films. Vaginal films can be intended for immediate or controlled release by optimizing the polymeric composition of the film, using different types of films, or combining thin film technology with other drug delivery strategies. Thin film dosage forms can be used for delivery of more than one active agent simultaneously and combination with other delivery strategies. Information is required to develop products that may play important role in the success of microbicide. A model for mucosal irritation and an understanding of the histological implications of drug products are required [94]. For vaginal administration, there should be better understanding of hydration and dissolution in limited volumes (e.g., 1 mL) of vaginal fluids. Selection of proper API is crucial in understanding of the therapeutic action of the drug. Usage of an applicator, applicator design for films, and determining how an applicator will be cleaned will be essential elements for the successful development of multiuse applicators.  Table 6 shows the different microbicidal vaginal formulations to enhance the efficacy of the incorporated drug as well as inhibiting the infections [95].

## 7. Challenges in Microbicide Development

Effective microbicide is not yet available; failures at different levels of their development leave a moot chat behind their outcome. Now is the time to understand where the lacking is and why? After having an effectual data in preclinical phases we are failing ultimately. Numerous candidates that had already been discussed possess some sort of similar unsuccessful story. If we search the reason behind, the conclusion will point toward some very genuine and mutual escapes.  Table 7 shows the outlook behind the disappointing microbicide strategy.

## 8. Nanotechnology Impact

Table 7 provides a broad idea regarding the loopholes that might be responsible for such fallouts. Every discussion points towards the need for the design of unconventional strategies for the topical vaginal microbicides. While exploring new options, the nanotechnology based microbicides and nanomicrobicides appear to be the best area. Various advances of this area comprise improvement of the local microbicide pharmacokinetic, better targeting, innovative barrier approaches, and options for combination therapy with decreased chance for resistance development. Various approaches were proposed and studies showed their potential role in advancing this preexposure prophylaxis. With more précised and finer targets, nanomicrobicides are the real hope for an efficient microbicidal product [96]. Here we are discussing some of the nanotechnology based approaches and their advancements.

### 8.1. Nanoparticle Based Microbicides

Nanoparticles provide a delivery strategy for targeted or controlled delivery to the vagina using topical microbicides. Three classes including drug containing polymeric nanoparticles, ligand targeted nanoparticles, nanoparticles that itself possess microbicidal ability are among the investigational candidates. CCR5 chemokine inhibitor protein PSC-RANTES loaded (PLGA) poly (lactic-co-glycolic acid) nanoparticles showed an increase tissue uptake, tissue permeation, and significant localization at the basal layers of the epithelium in an *ex vivo* cervical tissue model. But *in vivo* experiments in the rhesus macaque (macaca mulatta) SHIV model showed the need of higher dose of PSC-RANTES when compared with *in vitro* requirements [97]. They further observed the increased mucosal uptake of PSC-RANTES-loaded nanoparticles when compared to unformulated PSC-RANTES. Sustained gene silencing was observed in mice after vaginal delivery of siRNA carrying PLGA nanoparticles (<200 nm) mixed with the polyamines spermidine or putrescine. Polystyrene-based nanospheres with surface appended viral gp120 targeting lectin (concanavalin-A) showed their ability to arrest HIV-1 virions in *in vitro* model. Sensitivity of the nanoparticulate system can be modified in a required manner. pH responsive release of the microbicide in presence of human semen was observed from tenofovir loaded PLGA and eudragit S-100 copolymer nanoparticles. Over 72 hr there is a four-time upsurge in the release rate from 75% S-100 blend in the presence of semen fluid simulant [1]. In current times metallic nanoparticles are showing their role in this prophylaxis approach by virtue of their intrinsic anti-HIV activity. Silver nanoparticles with their complex mechanism exert size-dependent anti-HIV activity at an early stage of viral replication, most likely as a virucidal agent or as an inhibitor of viral entry. They bind to gp120 in a manner that prevents CD4-dependent viral binding, fusion, and infectivity, acting as an effective virucidal agent against cell free virus (laboratory strains, clinical isolates, T and M tropic strains, and resistant strains) and cell-associated virus [98]. PVP-coated silver nanoparticles showed to be a promising microbicide candidate as their formulation (replens gel) acts rapidly to inhibit HIV-1 transmission and offers long-lasting protection of the cervical tissue from infection for 48 h, with no indication of cytotoxicity observed in the explants. The ability of the mannose-coated gold nanoparticles to interfere with HIV-dendritic cells (DC) binding and distribution made them potential candidates to move with. Mannose residues have the ability to inhibit the binding between viral gp120 and DC-SIGN cell receptors and *α*-1,2-mannose disaccharide-coated gold nanoparticles have shown significant *in vitro* inhibition (85%–90%) of HIV-1 binding to DC-SIGN expressing cells and transinfection of T cells compared with *α*-1,2-mannose disaccharide alone [1].

### 8.2. Vesicular Systems

A liposome formulation was developed containing 1% octylglycerol and phosphatidyl choline in a ratio that revealed *in vitro* activity against neisseria gonorrhoeae, HSV-1, HSV-2, and HIV-1, while minimal toxicity was observed on vaginal *Lactobacillus* flora. *Ex vivo* testing in a human ectocervical tissue model or *in vivo* testing in the macaque safety model showed no sign of toxicity [98]. Delivery of antisense oligodeoxynucleotide ribozyme using pH-sensitive liposomes inhibited virus replication in monocyte-derived macrophage. Though, delivery of functional ribozymes by liposomes is relatively inefficient [99]. A new synthetic analogue of chemokine RANTES and −2 RANTES loaded commercial paucilamellar nonphospholipidic liposomes (novasomes 7474) was able to release this peptide based molecule in dose-dependent manner in *in vitro* conditions. Local safety studies done on murine and rabbit showed no evidence of cervicovaginal toxicity. Surprising results were seen during a further study on cynomolgus macaques (macaca fascicularis) challenged vaginally with R5-tropic SHIV162P3, where both either blank or drug loaded novasomes exhibited substantial prophylactic effect against infection, compared with the poor results of −2 RANTES in PBS. Results points towards the possible role of physical-chemical properties of these nanosystems and the importance of comprehensive mucosal coverage provided by novasomes for the observed protection against vaginal HIV transmission [100].

## 9. Preclinical and Clinical Studies on Microbicidal Products

The main aim of these studies is the evaluation of the activity and toxicity of active agents in animal as well as human being. Evaluate the effective dose concentrations, suitable route, side effects, efficiency, and stability of final products [101–104].  Table 8 shows the different studies which are carried out on microbicidal products.  Table 9 represents new preclinical microbicide candidate along with their mechanism of action.

## 10. Conclusion

Microbicide research is undergoing a period of rapid evolution. Development of safe and effective microbicides in developing countries promises to be one of the great public health concerns. Once developed these microbicides will be one of the crucial elements in any comprehensive response to HIV. Critical step will be to develop products that do not have to be used in a coastally dependent fashion. Microbicides will not only be integral to improving women's health but also will help reduce the burden of death and disease in women and eradicate poverty in the developing world.

## Figures and Tables

**Table 1:** Key events and predictions in the development of microbicides.

Year	Key events and predictions in the development of microbicides
In 1981	Acquired immunodeficiency syndrome (AIDS) was identified.
In 1983	Human immunodeficiency virus (HIV) was defined as causative agent.
In 1985–1988	Nonoxynol-9 was reported to destroy HIV *in vitro*.
In 1992	Nonoxynol-9 was applied vaginally in the macaque monkey to reduce the risk of infection.
In 1994	International working group on microbicides established Contraceptive Research and Development Program (CONRAD), US Centers for Disease Control and Prevention, US Food and Drug Administration, and US National Institute of Allergy and Infectious Diseases to facilitate global coordination of microbicide development.
In 1997	Nonsurfactant class of microbicides was shown to be safe and acceptable after phase I clinical trials in female volunteers.
In 1998	Phase III trials of nonoxynol-9 show no protection against HIV.
In 2000	First major international conference was devoted to microbicides.
In 2001	Promising microbicides entered phase II clinical trials to verify safety and acceptability.
In 2002	WHO report on nonoxynol-9 concludes that nonoxynol-9 should not be used for HIV/STI prevention.
In 2003-2004	Microbicides expected to enter phase III trials for effectiveness against HIV.
In 2007	First-generation microbicides showed; 50–60% effective against HIV.
In 2008	Researchers discovered that long chain polyanionic compounds could prevent *in vitro* HIV infection by preventing HIV entry into target cells through nonspecific inhibition of viral binding.
In 2010	Acid buffering gels could be used to lower vaginal pH and inactivate HIV and were shown to be safe in clinical trials.
In 2012-13	CAPRISA 008, a planned follow-on study to CAPRISA 004, which would evaluate the effectiveness of distributing 1% tenofovir gel in communities where CAPRISA 004 took place, was launched in 2012. Results from the facts 001 trial for 1% tenofovir gel are expected in 2013.

**Table 2:** Mechanism of action of different generations of microbicide candidates.

Mechanism of action	Candidates
First-generation microbicide (GEN-1)	
Viral disrupting agents	C-31G (savvy), nonoxynol-9, octoxynol-9, benzalkonium chloride, octyl glycerol/milk lipids, polybiguanides, sodium dodecyl sulphate, Z-14 (acylcarnitine analogue), and so forth.

Second-generation microbicide (GEN-2)	
Blocking HIV binding	Carrageenan, cellulose sulfate, naphthalene sulfonate, PRO 2000/5, dextrin-2-sulfate, heparan sulfate/cholic acid, polyanionic Gp120 inhibitors, polystyrene sulfonate, and so forth.

New-generation microbicide	
gp120-binding agents	mAb b12, CD IgG2, and BMS-806
Gp41-binding agents	T-20 (enfuvirtide), mAb 2F5, 4E10, and T1249
Targeting soluble CD4 receptor	mAb (TNX-355), soluble CD4-IgG
Targeting CXCR4 coreceptor	AMD3100, AMD070 small molecules antagonists
Targeting CCR5 coreceptor	PRO-140, PSC-RANTES
Dendritic cell uptake inhibitor	DC-SIGN and macrophage mannose binding receptor
NNRTI (nonnucleoside reverse transcriptase inhibitors)	MIV-150, TMC120, UC781, and S-DABO
NtRTIs (nucleotide reverse transcriptase inhibitors)	PMPA (tenofovir)
Integrase inhibitors	S-1360, C-731

**Table 3:** Ideal characteristics/attributes of microbicide development.

Commendable qualities	Attributes
Safety	(i) Should not exhibit any localized toxicity.
	(ii) Avoiding any potential impact on epithelial surfaces and natural innate barriers.
	(iii) Prevent from long-term systemic toxicity associated with frequency and duration of product.
	(iv) Avoiding impact on fertility and/or not exhibiting any fetal abnormalities.

Efficacy	(i) Product must have a significant degree of efficacy.
	(ii) Exhibit long-term efficacy.
	(iii) Do not produce drug resistance.

Cost	(i) Must be affordable to at-risk populations.
	(ii) They are cheap/affordable for mass distribution.

Acceptability	(i) Must be acceptable for use in conjunction with sex.
	(ii) Highly acceptable in the real world and majorly adopted by at-risk populations.

Drug delivery	(i) Sufficient drug levels must be maintained in the appropriate compartments of the genital tract or rectum during exposure to virus.
	(ii) Should exhibit sustained and controlled drug delivery to the target place.

Therapy impact	(i) Should not induce drug resistance.
	(ii) Exhibit significant impact of treatment and prevention of infection.

Prioritization	(i) Prioritization should include *in vitro* activity (potency and breadth), stage of product development (including manufacturing processes), and stability under diverse environmental conditions.
	(ii) Must be prioritized to maximize progress and prevent duplication.

**Table 4:** List of mucoadhesive polymers along with their bioadhesiveness.

Mucoadhesive polymers	Bioadhesiveness
CMC sodium	Excellent bioadhesiveness (+++)
Carbopol 934	Excellent bioadhesiveness (+++)
Polycarbophil	Excellent bioadhesiveness (+++)
Poly(acrylic acid/divinylbenzene)	Excellent bioadhesiveness (+++)
Sodium alginate	Excellent bioadhesiveness (+++)
Hydroxy ethyl cellulose	Excellent bioadhesiveness (+++)
HPMC	Excellent bioadhesiveness (+++)
Gelatin	Good bioadhesiveness (++)
Carrageenan	Fair bioadhesiveness (+)
PVA	Fair bioadhesiveness (+)
Chitosan	Fair bioadhesiveness (+)

**Table 5:** Factors affecting the bioadhesiveness of mucoadhesive polymers.

Factors	Effects on the bioadhesiveness of mucoadhesive polymers
Concentration of polymer	(i) Optimum concentration of a bioadhesive polymer produces maximum bioadhesion.
	(ii) In highly concentrated systems, the adhesive strength drops because the coiled molecules become separated from the medium so that the chains accessible for interpenetration become limited [9].

Molecular weight of polymer	(i) The threshold required for successful bioadhesion is at least 100,000 molecular weight.
	(ii) For example, polyethylene glycol (PEG), with a molecular weight of 20,000, has little adhesive character, whereas PEG with 200,000 molecular weight has improved, and a PEG with 400,000 has superior adhesive properties [10].

Flexibility	(i) Flexibility of mucoadhesive polymer is the key for interpenetration and entanglement.
	(ii) When water-soluble polymers become cross-linked, mobility of polymer chains decreases and thus decreasing the effective penetration into the mucus layer, which finally reduces bioadhesive strength [11].

pH	(i) pH can influence the formal charge on the surface of mucus as well as certain ionizable bioadhesive polymers.
	(ii) pH of the medium is chief factor for the degree of hydration of crosslinked polymers, showing every time increased hydration from pH 4 to 7 and then a decrease as alkalinity and ionic strength increases [12].

Swelling	(i) It mainly depends on the polymer concentration, ionic concentration, and the presence of water.
	(ii) Formation of slippery mucilage without adhesion after overhydration [13].

Contact time	(i) Contact time between the bioadhesive and mucus layer determines the extent of swelling and interpenetration of the bioadhesive polymer chains.
	(ii) Moreover, bioadhesive strength increases as the initial contact time increases [14].

Mucin turnover	(i) The mucin turnover is anticipated to limit the residence time of the mucoadhesive on the mucus layer.
	(ii) Mucoadhesive polymers are detached from the surface due to mucin turnover [15].

Disease states	The physiochemical properties of mucus are known to change during disease conditions such as bacterial and fungal infections of the female reproductive tract [16].

**Table 6:** Drug incorporated microbicidal vaginal formulations.

Polymers	Drug (dosage form)	Results
Sodium carboxymethyl cellulose	Dapivirine (vaginal tablet)	Developed as a vaginal microbicide and improved wettability of the drug [17].

Polycarbophil, carbopol 974P	Tenofovir disoproxil fumarate (vaginal tablet)	Assess acceptability, safety, and effectiveness in preventing HIV infection [18].

Polymethacrylate salt	Tenofovir (vaginal solution)	Controlled microbicide delivery template by intravaginal route for HIV prevention [19].

Propylene glycol caprylate	UC-781(vaginal emulsion)	Enhancing the vaginal absorption of these microbicidal candidates [20].

Chromophore EL-capryol 90, tween 80	Fluconazole (FLZ) (vaginal emulsion)	Showed significantly higher (*P* < 0.05) *in vitro* bioadhesion and antifungal activity without any vaginal irritation in rabbit [21].

Carbopol, tween 80	Nitro imidazole, ornidazole (vaginal emulsion)	A high release was found in the alkaline pH and locally effective in vagina [22].

Phospholipon 90G, captex 300, pluronic F68, and Cremophor EL	WHI-07, vanadocene dithiocarbamate (vaginal emulsion)	Demonstrated that WHI-07 either alone or in combination with a vanadocene has clinical potential for the development of a dual-function anti-HIV microbicide for sexually active women [23].

Tragacanth gum, aacacia gum	Miconazole nitrate (vaginal suspension)	Exhibit uniformly distribution of drug and show locally effect to control the infection [24].

Polycarbophil, carbopol 974P	Tenofovir (TFV), IQP-0528 (vaginal gel)	These gels have the potential for dual compartment use to inhibit the virus entry [25].

Pluronic	Metronidazole (vaginal gel)	Successfully used for treatment of bacterial vaginosis [26].

Cellulose acetate phthalate (CAP)	Model drug (vaginal cream)	Use as a topical microbicide for preventing HIV-1 infection in humans [27].

Hydroxyethyl cellulose	Tenofovir (vaginal gel)	Exhibit excellent effectiveness in preventing human immunodeficiency virus transmission [28].

Hydroxyethyl cellulose	Tenofovir (vaginal ring)	Exhibit potential for the prevention of transmission of (HIV-1) in pig-tailed macaques [29].

Polyurethane	Pyrimidinedione IQP-0528 (PYD1) IQP-0532 (PYD2) (vaginal ring)	Prophylactic drug delivery systems to prevent the sexual transmission of HIV-1 [30].

Polyether urethanes	Dapivirine(vaginal ring)	Sustained delivery of microbicidal agents [31].

Silicone elastomer	TMC120 (vaginal ring)	Controlled release strategy for delivering microbicidal substances for the prevention of heterosexual transmission of HIV [32].

Polyvinyl alcohol(PVA)	Pyrimidinedione, IQP-0528 (vaginal films)	Nontoxic in nature and exhibit excellent prevention against HIV infection [33].

Octylglycerol	Dapivirine, tenofovir,UC781 (vaginal films)	Prevent HIV infection and products are nontoxic to the endogenous vaginal *Lactobacillus* [34].

Span 60 or span 40, cholesterol	Nystatin (NYS) (vaginal films)	Reducing its toxicity and making it a more active against vaginal infections [35].

Polyvinyl alcohol (PVA)	Retrocyclin analog RC-101 (vaginal films)	Shown to maintain bioactivity for a period of 6 months [36].

Hydroxypropyl cellulose, xanthan gum	Clindamycin (CL) phosphate (vaginal films)	Exhibit potential vaginal delivery system of CL against bacterial vaginosis [37].

Hydroxypropyl cellulose/polyethylene glycol 400	Itraconazole (vaginal films)	Exhibit more effective treatment against vaginal candidiasis and do not affects normal vaginal flora [38].

Cellulose acetate 1,2-benzenedi carboxylate	Octoxynol-9 or nonoxynol-9 (vaginal films)	Exhibit biocompatibility of solid-dosage forms of anti-HIV virus type 1 microbicides with the human cervicovaginal mucosa modeled *ex vivo* [39].

**Table 7:** Outlook behind the disappointing microbicide strategy.

Prerequisite of microbicide candidates	Probable mechanism	Reason for the failure
Candidates who require coating the vaginal epithelial lining uniformly.	Barrier for invasion and disruption of virus	Inability of the delivery system to provide an effective and durable microbicide fence along the epithelial lining.

Candidates who require retaining at the site to achieve appropriate concentration levels either in local tissues or blood.	Reverse transcriptase inhibitor, integrase inhibitor, and protease inhibitor	Insufficiency of delivery systems to provide sustained and controlled microbicide release.

Candidates who require targeting to a particular site	Entry inhibitors (targeting viral and host cell receptors).	Inability of delivery process to target active molecule to required site.

**Table 8:** Preclinical and clinical studies on microbicidal products.

Studies	Aim of study	Time period
Preclinical studies	(i) Screening and testing in laboratory.	1–10 years
	(ii) Animal studies for the evaluation of the activity and toxicity of active agents.	

Phase I clinical trials	(i) Early testing in small groups of 10–20 human volunteers.	2-3 years
	(ii) Confirming the lack of toxicity and the delivery of effective doses.	

Phase II clinical trials	Larger phase II trials are done in many hundreds or up to a few thousands of volunteers to obtain effectiveness data for promising candidates.	2–5 years

Phase III clinical trials	(i) To demonstrate effectiveness, safety, and acceptability in thousands of human volunteers involves large-scale testing.	2–6 years
	(ii) It is providing statistically significant data for review by regulatory agencies (e.g., the U.S. FDA or others) before new products can be approved for marketing and use.	

Phase IV study	For microbicides, postmarketing surveillance might also include any long-term effects on HIV disease progression and treatment (including possible selection of drug-resistant HIV when relevant), HIV risk behavior, and interactions with other diseases, therapies, or products.	Indefinitely

Other preapproval studies	(i) Data from additional studies are often required by regulatory agencies for the approval of new products.	2–6 years
	(ii) Regulatory agencies also require information regarding product manufacturing methods and quality control measures to ensure that the marketed product is the same as the tested product.	

Product introduction and supporting studies	Addressing policy and logistical issues is often the key for the introduction of new health products. For microbicides, many countries will require preintroductory studies in their own populations before allowing the importation and distribution of a new product.	up to 10 years

**Table 9:** New preclinical microbicide candidates with their mechanism of action.

Mechanism of action	Candidates
Vaginal defense enhancer	Genetically engineered probiotics, mucocept HIV, RANTES peptides, and *Lactobacillus* delivered cyanovirin-N

Entry/fussion inhibitors	Aptamers, betacyclodextrin, flavinoids, porphyrins, siRNA, silver nanoparticles, ISIS 5060, reterocyclines, and TatCD

Combination approaches	Buffer gel with dendrimers, M167, BMS, and other ARV

Microbicide combined with devices	Duet cervical barrier and condoms with alkyl sulfate coating

Uncharacterized mechanism	CO (ciclo piroxolamine)

Surfactants	Alkyl sulfates (surfactants and chaotropic agents)
